# Comparative effectiveness of angioembolization versus open surgery in patients with blunt splenic injury

**DOI:** 10.1038/s41598-024-59420-w

**Published:** 2024-04-16

**Authors:** Toshinao Suzuki, Atsushi Shiraishi, Kensuke Ito, Yasuhiro Otomo

**Affiliations:** 1 Department of Anesthesiology, Kimitsu Chuo Hospital, Chiba, Japan; 2 Emergency and Trauma Center, Kameda Medical Center, 929 Higashicho, Kamogawa, Chiba 296-8602 Japan; 3 Interventional Radiology Center, Teikyo University Chiba Medical Center, Chiba, Japan; 4 Trauma and Acute Critical Care Center, Medical Hospital, Tokyo Medical and Dental University, Tokyo, Japan; 5 National Disaster Medical Center, Tokyo, Japan

**Keywords:** Medical research, Health care, Surgery

## Abstract

The effectiveness and safety of transcatheter splenic artery embolization (SAE) compared to those of open surgery in patients with blunt splenic injury (BSI) remain unclear. This retrospective cohort-matched study utilized data from the Japan Trauma Data Bank recorded between 2004 and 2019. Patients with BSI who underwent SAE or open surgery were selected. A propensity score matching analysis was used to balance the baseline covariates and compare outcomes, including all-cause in-hospital mortality and spleen salvage. From 361,706 patients recorded in the data source, this study included 2,192 patients with BSI who underwent SAE or open surgery. A propensity score matching analysis was used to extract 377 matched pairs of patients. The in-hospital mortality rates (SAE, 11.6% vs. open surgery, 11.2%, adjusted relative risk (aRR): 0.64; 95% confidence interval [CI]: 0.38–1.09, *p* = 0.10) were similar in both the groups. However, spleen salvage was significantly less achieved in the open surgery group than in the SAE group (SAE, 87.1% vs. open surgery, 32.1%; aRR: 2.84, 95%CI: 2.29–3.51, *p* < 0.001). Survival rates did not significantly differ between BSI patients undergoing SAE and those undergoing open surgery. Nonetheless, SAE was notably associated with a higher likelihood of successful spleen salvage.

## Introduction

The spleen is the most commonly injured abdominal organ after blunt trauma. Until the 1960s, splenectomy stood as the primary treatment for blunt splenic injury (BSI). However, over the past few decades, there has been a significant shift in the management of BSI, favoring non-operative approaches. Splenic angioembolization (SAE) has become increasingly common as a standard management in patients with BSI^1–6^. Theoretically, SAE can provide a less invasive intervention to ensure hemostasis and preserved splenic function in comparison with open splenic surgery^7^. While open surgery is preferred as the standard treatment in hemodynamically unstable patients with BSI, SAE has been reported to be as effective and safe as open splenic surgery for controlling hemorrhage^8–10^.

The effectiveness and safety of SAE compared to open splenic surgery have been poorly reported by limited studies, and these retrospective, non-randomized designs without adjustment for confounders, might inevitably have led to bias^11–14^. The current study aimed to compare the clinical outcomes between SAE and open splenic surgery after adjustment for known covariates in patients with BSI.

## Results

**Figure 1:**
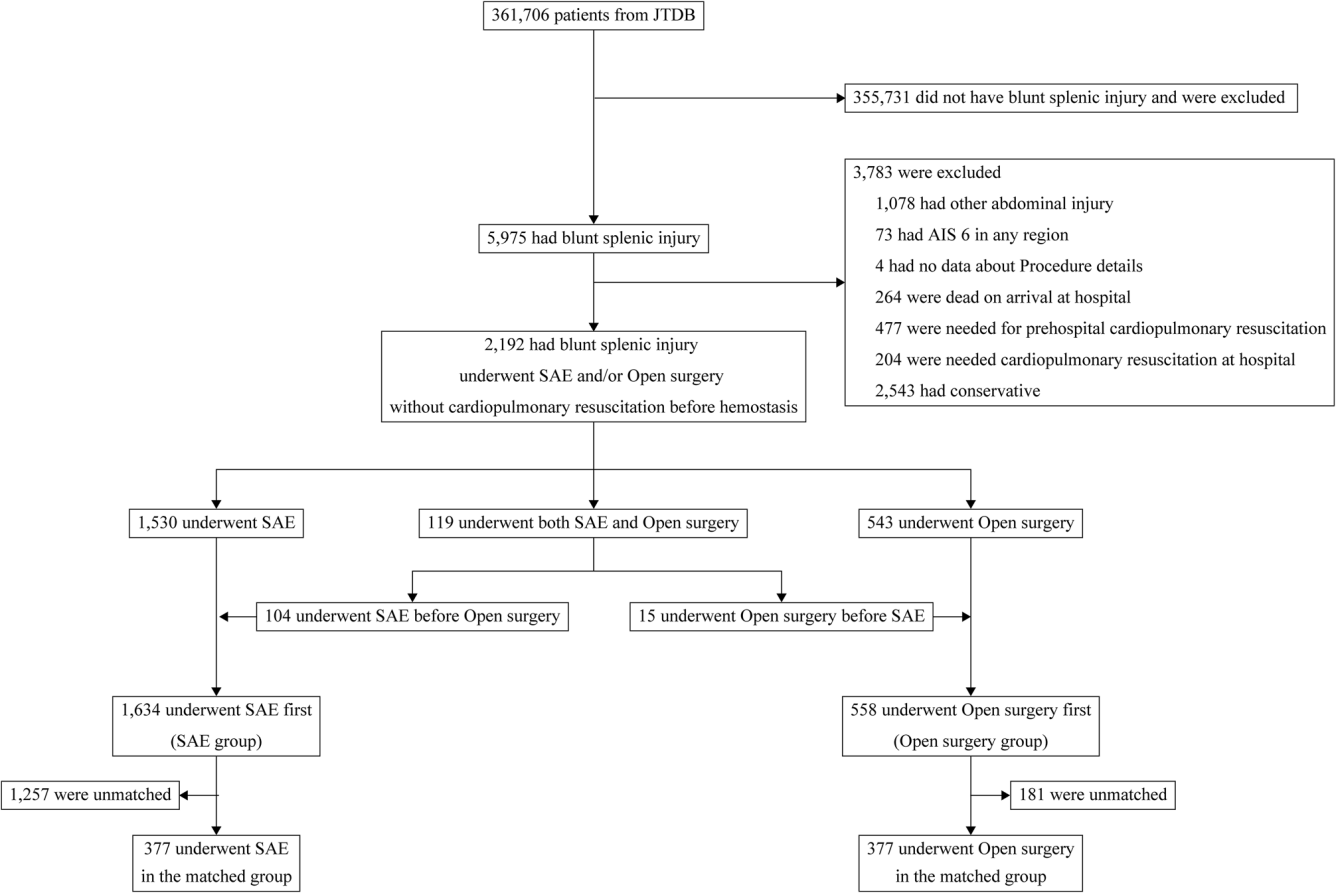
Study participant selection. AIS, Abbreviated Injury Scale; JTDB, Japan Trauma Data Bank; SAE, splenic artery embolization.

Among the 361,706 trauma patients registered in the Japan Trauma Data Bank (JTDB), 2,192 patients with BSI who underwent hemostatic treatment were identified (Fig. 1). After assignment to the treatment groups according to the procedure that the patients underwent first, the SAE and open surgery groups comprised 1,634 (74.5%) and 558 patients (25.5%), respectively. The SAE group included 104 patients (6.4%) who underwent open surgery after SAE, whereas the open surgery group included 15 patients (2.7%) who underwent SAE after open surgery. In-hospital deaths occurred in 93 (5.8%) and 64 (11.9%) patients in the SAE and open surgery groups, respectively.

**Figure 2:**
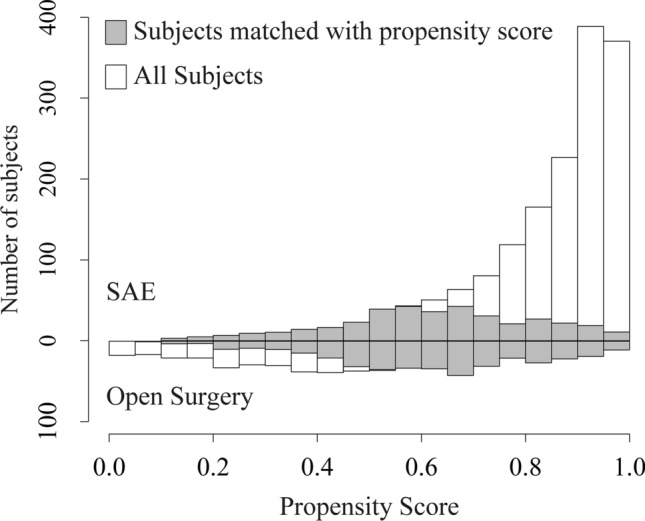
Histograms showing the density of propensity score distributions in the groups before and after matching. SAE, splenic artery embolization.

The propensity score matching selected 377 pairs of patients who initially underwent SAE or open surgery and whose baseline covariates were well balanced except for airway management and blood transfusion (Tables 1 and 2). This cohort of patients comprised 23% and 68% of patients who underwent SAE and open surgery, respectively, before the matching (Fig. 2).

**Table 1:** Baseline characteristics before and after propensity score-based matching.

	Before matching			After matching		
	SAE	Open surgery	SMD	SAE	Open surgery	SMD
Patients	1,634	558		377	377	
Sex, female, n (%)	470 (28.8)	123 (22.0)	0.155	80 (21.3)	93 (24.8)	0.081
Age, in years, (SD)	42 (23)	42 (22)	0.033	43 (23)	42 (22)	0.040
Vital signs at hospital arrival						
Systolic blood pressure, mmHg, (SD)	114 (29)	102 (32)	0.380	102 (30)	105 (32)	0.074
Heart rate, beats/min, (SD)	95 (25)	99 (27)	0.160	100 (26)	98 (26)	0.066
Body temperature, °C, (SD)	36.3 (0.9)	36.0 (1.1)	0.297	36.0 (1.1)	36.1 (1.0)	0.061
GCS, median [IQR]	15 [14–15]	14 [12–15]	0.298	14 [12–15]	14 [13–15]	0.110
Medical history, n (%)						
Coronary heart disease	33 (2.0)	9 (1.6)	0.030	7 (1.8)	6 (1.6)	0.019
Congestive heart failure	15 (0.9)	6 (1.1)	0.016	5 (1.2)	3 (0.8)	0.044
Hypertension	171 (10.5)	54 (9.7)	0.026	33 (8.7)	34 (8.9)	0.01
Stroke	33 (2.0)	11 (2.0)	0.003	7 (1.8)	5 (1.3)	0.042
Dementia	20 (1.2)	9 (1.6)	0.033	7 (1.9)	5 (1.3)	0.046
Chronic obstructive pulmonary disease	12 (0.7)	1 (0.2)	0.082	2 (0.4)	1 (0.3)	0.017
Peptic ulcer	13 (0.8)	6 (1.1)	0.029	3 (0.9)	4 (1.1)	0.02
Liver cirrhosis	15 (0.9)	10 (1.8)	0.076	6 (1.6)	4 (1.1)	0.046
Diabetes mellitus	89 (5.4)	34 (6.1)	0.028	25 (6.6)	23 (6.0)	0.022
Chronic renal failure	19 (1.2)	3 (0.5)	0.068	1 (0.3)	2 (0.5)	0.044
Malignancy	16 (1.0)	6 (1.1)	0.010	2 (0.6)	2 (0.5)	0.011
Hematological disease	2 (0.1)	3 (0.5)	0.072	2 (0.5)	1 (0.3)	0.042
Human immunodeficiency virus	1 (0.1)	0 (0.0)	0.035	0 (0.0)	0 (0.0)	< 0.001
AIS code for splenic injury, n (%)						
NFS(544,299.2)	68 (4.2)	18 (3.2)	0.05	16 (4.3)	15 (4.1)	0.011
Contusion						
NFS (544,210.2)	44 (2.7)	7 (1.3)	0.104	5 (1.4)	6 (1.6)	0.018
Minor, superficial; OIS I, II (544,212.2)	172 (10.5)	28 (5.0)	0.207	21 (5.5)	25 (6.6)	0.048
Major; OIS III (544,214.3)	174 (10.6)	30 (5.4)	0.195	25 (6.5)	29 (7.6)	0.043
Laceration						
NFS (544,220.2)	23 (1.4)	9 (1.6)	0.017	7 (1.8)	6 (1.6)	0.016
Minor, superficial; OIS I, II (544,222.2)	98 (6.0)	37 (6.6)	0.026	29 (7.8)	29 (7.7)	0.005
Moderate; OIS III (544,224.3)	630 (38.6)	136 (24.4)	0.309	92 (24.3)	113 (29.9)	0.125
Major; OIS IV (544,226.4)	352 (21.5)	198 (35.5)	0.313	140 (37.1)	124 (32.9)	0.087
Massive; OIS V (544,228.5)	42 (2.6)	63 (11.3)	0.349	25 (6.7)	18 (4.7)	0.089
Rupture						
NFS (544,240.3)	43 (2.6)	35 (6.3)	0.177	20 (5.3)	15 (4.1)	0.056
Injury Severity Score*, median [IQR]	22 [13–34]	25 [16–36]	0.246	27 [17–36]	25 [16–36]	0.130
AIS body region, median score [IQR]						
AIS 1: head	0 [0–1]	0 [0–1]	0.075	0 [0–2]	0 [0–1]	0.032
AIS 2: face	0 [0–0]	0 [0–0]	0.008	0 [0–0]	0 [0–0]	0.013
AIS 3: neck	0 [0–0]	0 [0–0]	0.090	0 [0–0]	0 [0–0]	0.001
AIS 4: thorax	3 [0–4]	3 [0–4]	0.091	3 [0–4]	3 [0–4]	0.041
AIS 5: abdomen*	3 [3–3]	3 [3–4]	0.466	3 [3–4]	3 [3–4]	0.094
AIS 6: spine	0 [0–0]	0 [0–0]	0.030	0 [0–0]	0 [0–0]	0.025
AIS 7: upper extremity	0 [0–1]	0 [0–1]	0.064	0 [0–1]	0 [0–1]	0.02
AIS 8: lower extremity	0 [0–2]	0 [0–2]	0.089	0 [0–2]	0 [0–2]	0.005
AIS 9: unspecified	0 [0–0]	0 [0–0]	0.046	0 [0–0]	0 [0–0]	0.027

The variables listed in the table were used to estimate the propensity score to predict the likelihood of undergoing SAE. Categorical and continuous variables are expressed as absolute counts (%) and mean (SD), respectively, unless otherwise specified. GCS and AIS body region are expressed as median IQR.

AIS, Abbreviated Injury Scale; GCS, Glasgow Coma Scale; IQR, interquartile range; NFS, not further specified; SD, standard deviation; OIS, Organ Injury Scale; SMD, standardized mean difference.

*Not included in variables to estimate propensity score.

**Table 2:** Resuscitative management before and after propensity score-matching.

Procedures performed in the trauma bay	Before matching			After matching		
	SAE N = 1,634	Open surgery N = 558	SMD	SAE N = 377	Open surgery N = 377	SMD
Airway management, n (%)	512 (31.3)	275 (49.3)	0.372	194 (51.5)	166 (44.1)	0.148
Chest drain	293 (17.9)	146 (26.2)	0.200	106 (28)	95 (25.1)	0.065
Vasopressor administration	88 (5.4)	67 (12.0)	0.237	46 (12.2)	41 (10.8)	0.044
Aortic occlusion	46 (2.8)	50 (9.0)	0.263	29 (7.7)	25 (6.5)	0.049
Blood transfusion	614 (37.6)	314 (56.3)	0.381	210 (55.7)	186 (49.4)	0.127

All variables listed in the table were used to estimate the propensity score to predict the likelihood of undergoing SAE. Resuscitation management is an important step needed to correct physiological disorders that must be performed in trauma bays. Categorical variables are expressed as absolute counts (%).

SAE, splenic artery embolization; SMD, standardized mean difference.

After the matching, among 377 patients in the open surgery group, 258 (68.4%) underwent total splenectomy, while 119 (31.6%) underwent splenic suture or partial resection. Among these 119 patients, SAE was additionally performed in 13 individuals. In the SAE group, 56 patients (14.8%) underwent additional surgeries, with total splenectomy performed in 48 patients. Patients in both groups were considered to be severely injured based on the median Injury Severity Score (SAE, 27 [interquartile range (IQR): 17–36] vs. open surgery, 25 [IQR: 16–36]).

**Figure 3:**
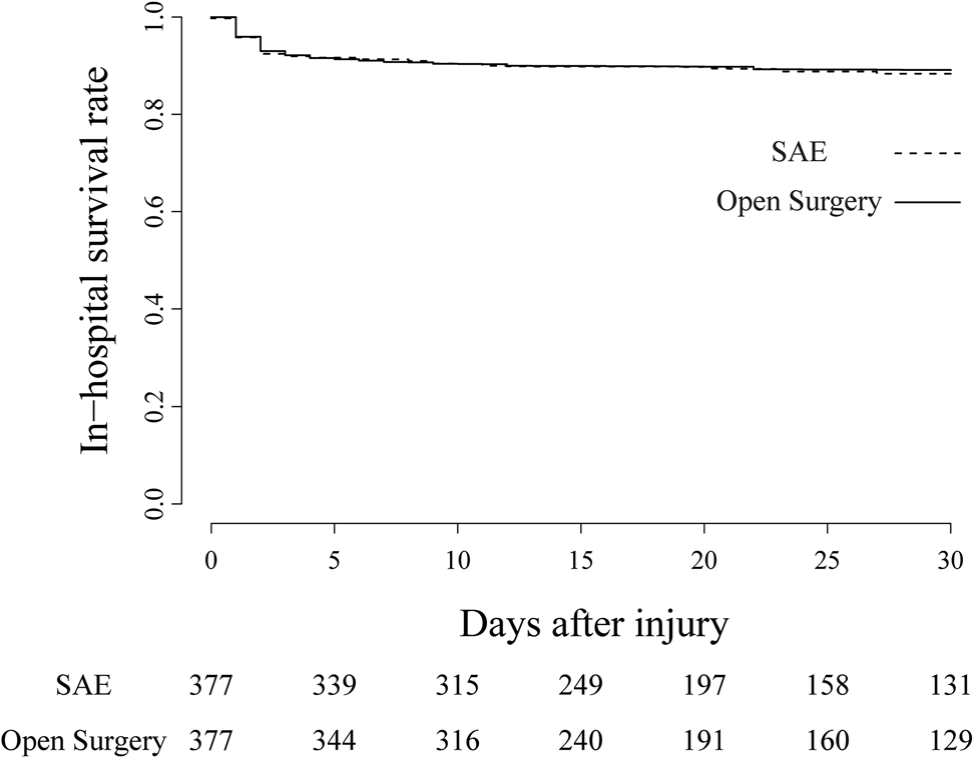
Kaplan–Meier curves for in-hospital survival. Kaplan–Meier curves for in-hospital survival among patients with isolated blunt splenic injury based on the use of open surgery or splenic artery embolization. SAE, splenic artery embolization.

**Figure 4:**
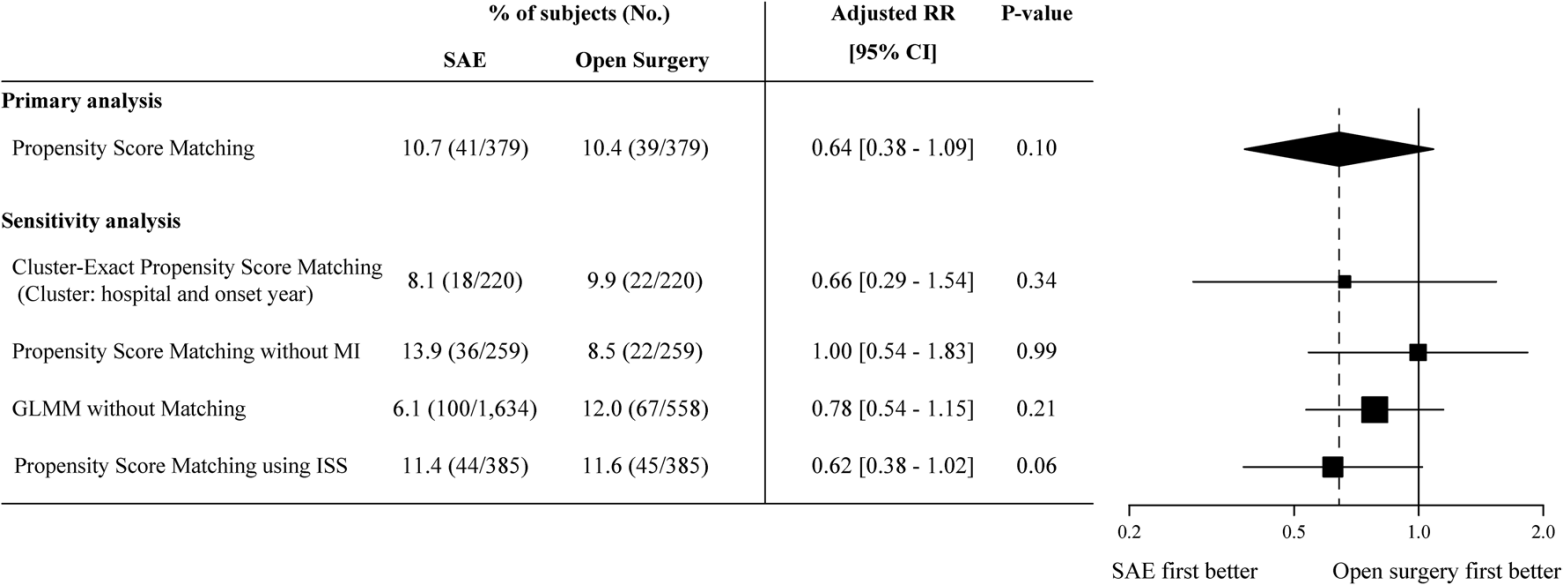
Primary analysis and sensitivity analysis of in-hospital mortality. The primary analysis was a propensity score matching that used the multiply-imputed dataset. The first sensitivity analysis was a cluster-exact propensity score matching that select 1:1 matched pairs of patients treated in the same institution and in the same injury period, to account for disparities between facilities. The second sensitivity analysis was a propensity score matching that used the naïve dataset without multiple imputation to test the robustness of the multiple imputations. The third sensitivity analysis tested the generalized linear mixed effects model used with the multiply-imputed datasets but without matching, to test the robustness of the propensity score matching. The other sensitivity analysis was a propensity score matching that used the Injury Severity Score for matching instead of the AIS score, and the analysis method itself was the same as the primary analysis. Outcomes were analyzed using the double-adjustment method. CI, confidence interval; ISS, Injury Severity Score; GLMM, generalized linear mixed effects model; MI, multiple imputation; OR, odds ratio; SAE, splenic artery embolization.

The in-hospital mortality was similar for the two matched groups (SAE, 44 [11.6%] vs. open surgery, 42 [11.2%]; unadjusted relative risk (RR): 1.03; 95% confidence interval (CI): 0.67–1.59; adjusted RR [aRR]: 0.64; 95%CI: 0.38–1.09). The likelihood of spleen salvage was significantly higher in the SAE group than in the open surgery group (SAE, 327 [87.1%] vs. open surgery, 121 [32.1%]; unadjusted RR: 2.71; 95%CI: 2.37–3.10; adjusted RR: 2.84; 95%CI: 2.29–3.51). There were no significant differences between the SAE and open surgery groups in terms of survival time (Fig. 3), hospital-free days at day 28, abdominal complications, subsequent organ failure, infectious complications, or central nervous system complications (Table 3). All the sensitivity analyses showed nearly consistent results to those of the primary analysis (Fig. 4, Tables E1, E2, E3, E4, E5, E6 and E7).

**Table 3:** Study outcomes.

Outcomes	SAE N = 377	Open surgery N = 377	Relative risk or difference		*P* value
			Unadjusted	Adjusted	
In-hospital mortality, n (%)	44 (11.6%)	42 (11.2%)	1.03 [0.67–1.59]	0.64 [0.38–1.09]	0.10
Spleen salvage, n (%)	327 (87.1%)	121 (32.1%)	2.71 [2.37–3.10]	2.84 [2.29–3.51]	< 0.001
Hospital-free days at day 28, median [IQR]	1 [0, 13]	2 [0, 14]	0 [− 1 to 1]	0 [− 1 to 1]	0.74
Complications, n (%)					
Overall	76 (20.2%)	74 (19.7%)	1.02 [0.75–1.40]	0.88 [0.61–1.29]	0.50
Abdominal	29 (7.8%)	24 (6.3%)	1.23 [0.71–2.14]	1.06 [0.57–2.00]	0.83
Organ failure	38 (10.1%)	37 (9.8%)	1.02 [0.65–1.61]	0.84 [0.47–1.48]	0.54
Infection	35 (9.3%)	34 (9.1%)	1.02 [0.63–1.64]	0.85 [0.49–1.47]	0.67
CNS	14 (3.7%)	13 (3.4%)	1.07 [0.48–2.38]	0.86 [0.34–2.19]	0.90

Spleen salvage was defined as the avoidance of total splenectomy. The number of hospital-free days at day 28 was defined as the number of days that the patient was alive and not hospitalized during the first 28 days after the hospital admission. Hospital-free days at day 28 is expressed as median IQR. The definition of complications is described in Appendix E1. Data are reported as number (%) or relative risk/difference [95% confidence interval].

CNS, central nervous system; IQR, interquartile range; SAE, splenic artery embolization.

## Discussion

The likelihood to undergo SAE increased over time and varied across the study hospitals. This study used a propensity score matching analysis to adjust for imbalances in baseline patient characteristics and cluster effects, and further compared the outcomes after SAE or open surgery in patients with BSI. There were no significant differences in terms of in-hospital mortality and all the complications that were analyzed. However, the SAE group was significantly more likely to experience spleen salvage. The associations between selection of SAE or open surgery and in-hospital mortality were consistent in the sensitivity analyses.

Nonoperative management including SAE is increasingly preferred over surgical management of BSIs^15,16^. Several studies have reported that SAE increased the success rate for nonoperative management^6,15,17,18^. These preferences for SAE and associations of SAE and clinical outcomes varied over time and across hospitals^16,19^. In the sensitivity analysis, a cluster-exact propensity score matching using 2 cluster variable, including the hospital IDs and treatment period (2004–2012, and 2013–2019) as the cluster variable, demonstrated consistent association with the study primary analysis.

If the patient characteristics and indications for hemostasis are heterogeneous across study hospitals, direct comparisons between SAE and open surgery in patients with BSI might be inappropriate. In this study, the existence of a large number of unmatched populations could be a consequence of this heterogeneity. However, the likelihood to undergo SAE increased over time, and varied across the study hospitals. This shift to using SAE over time and across institutes suggests a presence of overlap in the indications for the procedures. Hence, this study did not estimate the average treatment effect in overall patients with BSI; rather, it estimated the average treatment effect for the population that is likely to undergo either SAE or open surgery.

The results of this study enabled the calculation of the sample size required for a randomized controlled trial to evaluate the non-inferiority of SAE to open surgery in patients with BSI. Based on a one-sided, normal-approximation, and a non-inferiority test at a 2.5% significance level, approximately 10,286 patients per group would be required to provide an 80% power for demonstrating that the upper limit of the 95%CI for the treatment difference was ≤ 2%, which was a prespecified non-inferiority margin for this outcome. If the upper limit of the 95%CI for the treatment difference was changed to 1% or 0% (superiority trial), the required sample sizes would be 60,505 patients and 329,196 patients, respectively. This study only included 377 matched pairs of patients, which was under the minimum number that was needed for confirming the efficacy of SAE for BSI. Further studies are needed to prospectively verify the current findings.

This study had some limitations. First, despite propensity score-based matching, the retrospective design predisposed the study to unidentified and unmeasured confounders. The retrospective design of this study also limited our ability to obtain clinical information and time course data about patients not registered in the database. Ideally, data related to the time course should have been used for grouping patients. However, since this information was unavailable, we had to group patients based on variables related to the registered hemostasis-related codes. In nature of an observational study, there is no time point at which the patients are randomized to SAE or laparotomy. It is possible that SAE and laparotomy differed in the time between the decision and the initiation of the procedure, which could lead to a survivorship bias. Nevertheless, it may be challenging to account for potential biases given the absence of time-dependent variables or decision-to-procedure time in the database. Second, the cause of mortality was not recorded. In comparing the observed mortality rate with that of the past, it is essential to interpret with caution due to the unrecorded causes of mortality. Third, the information regarding the specific treatment methods for both SAE and open surgery is insufficient. Fourth, the data source did not provide information on the exact location of abdominal angioembolization. As a result, in cases where abdominal angioembolization was performed on patients with multiple abdominal organ injuries, the target organ could not be identified accurately. Consequently, patients with multiple organ injuries in the abdominal region were inevitably excluded, and only patients with splenic injury who underwent angioembolization of the spleen were included. The selection of patients with only splenic injury might lead to selection bias, thereby limiting the generalizability of the results. The presence of non-splenic intra-abdominal injuries could largely increase the likelihood to undergo open surgery rather than SAE. Fifth, there was a possibility of intergroup heterogeneity because of the substantially higher number of patients who underwent SAE (1,634 patients) than the number of patients who underwent open surgery (558 patients). Thus, matching to the open surgery group might decrease the generalizability of the results. Finally, the matched analysis in this study had some unbalanced covariates. Nonetheless, double-adjustment was used to remove confounding for imbalance, that existed after propensity score matching.

## Conclusions

The in-hospital mortality did not significantly differ between Japanese patients with BSI, who underwent hemostatic management with either SAE or open surgery.

## Materials and methods

### Study design

A retrospective registry-based matching-cohort study utilized data from the JTDB recorded between April 2004 and March 2019. The JTDB is a nationwide trauma registry that was jointly established in 2003 and has since been maintained by the Japanese Association for the Surgery of Trauma and the Japanese Association for Acute Medicine. As of March 2019, a total of 280 hospitals participate in the JTDB. These hospitals voluntarily register trauma or burn patients whose injury severity was scored with an Abbreviated Injury Scale (AIS) of ≥ 3. The registered data include variables for demographic information, mechanism(s) of injury, pre-hospital treatments, physiological status at the scene and upon hospital arrival, comorbidities, in-hospital examinations and treatments, diagnosis based on the AIS coding, injury severity, and patient outcomes (Table E8).

Reporting of this study adhered to the Strengthening the Reporting of Observational Studies in Epidemiology (STROBE) statement^20^. Ethics approval was obtained from the committees of the Japanese Association for the Surgery of Trauma and each participating institution. This study was performed in accordance with the Declaration of Helsinki. Because anonymized data from the JTDB was used, the need for informed consent was waived in accordance with the Ethical Guidelines for Medical and Health Research Involving Human Subjects published by the Ministry of Health, Labor, and Welfare of Japan. The approval document from the Japanese Association for the Surgery of Trauma and the representative institution (National Defense Medical College Research Institute) are available on the JTDB website (https://www.jtcr-jatec.org/traumabank/dataroom/ethics2.htm) (approval ID No. 2548).

### Participants

This study included patients with BSI requiring hemostatic intervention. Patients diagnosed with BSI were coded using the AIS code specifying BSI (Table E9). Inclusion criteria were applicable to those who had undergone SAE or open surgery including suturing and partial or total resection of the spleen. The AIS 90 update 98 edition was used in this study. The JTDB lacked information where the target abdominal organ (liver, spleen, or kidney) of abdominal angioembolization was; therefore, only in patients with single abdominal organ injuries, this deficit inevitably limited the identification of organs that underwent abdominal SAE. Patients with multiple organ injuries in the abdominal region, and those with severe injury (AIS code ≥ 3) to abdominal organs other than the spleen (e.g., liver or kidney) were excluded (Table E10). Furthermore, the following patients were excluded from the study: patients who presented with cardiac arrest during trauma care in the prehospital or emergency room, had any of the AIS codes for an unsurvivable injury to any region of the body (AIS code = 6), or had missing data on variables for hemostatic procedures. The study inclusion and exclusion criteria were applied before the removal of outliers and multiple imputations. Patients were assigned to the SAE or open surgery group based on which hemostasis treatment they received first. The details are described in Appendix E1.

### Study outcomes

The study outcomes were all-cause in-hospital mortality, spleen salvage, hospital-free days at day 28, abdominal complications, organ failure, infectious complications, and central nervous system complications. Spleen salvage was defined as not undergoing total splenectomy. “Hospital-free days at day 28” was defined as the number of days that the patient was alive and not hospitalized during the first 28 days after arrival at the emergency department. The details of complications are described in Appendix E2.

### Statistical analysis

After applying the study selection criteria, outliers in the numeric study variables were removed using robust linear regression analysis. Furthermore, missing values in all the study variables were imputed using multiple imputation by chained equation (Appendix E3, Table E11). The current study utilized propensity score matching to adjust for the baseline difference of covariates across patients who had undergone SAE or open surgery. The propensity score was estimated by a generalized linear mixed effect model using patient-level covariates as fixed effect covariates and the study hospitals and study period as random effect covariates. The patient level covariates included characteristics, resuscitative procedures during the emergency department stay, injury severity, medical history, type of splenic injury, and blood transfusion as fixed-effect explanatory variables. Moreover, propensity to undergo SAE differed substantially across the hospitals (Fig. E1), and this difference increased over the study period (Fig. E2). Therefore, the hospital identifier and the year of injury (2004–2011 vs. 2012–2019) were regarded as random effect explanatory variables (Appendix E4). Propensity-score matching selected 1:1 matched pairs of patients who underwent SAE or open surgery.

The primary analysis was an intergroup comparison of the study outcomes and reported the estimated relative risk or absolute difference with the corresponding 95% confidence interval. These associations were calculated using a Poisson regression generalized linear mixed effects model that was again adjusted for fixed and random effect covariates, except for the year of injury, to clean up any residual intergroup differences in confounders after the matching^21^. Four sensitivity analyses were performed to test the robustness of the primary analyses. The details about sensitivity analyses are described in Appendix E5.

All statistical analyses were performed using the R software version 3.5.0 (R Foundation for Statistical Computing, Vienna, Austria). Continuous variables are presented as mean ± standard deviation or median interquartile range, whereas categorical variables are presented as percentages. Differences were considered significant at *P*-values of < 0.05.

### Ethics approval and consent to participate

Ethics approval was obtained from the committees of the Japanese Association for the Surgery of Trauma, as well as each participating institution. Following the Ethical Guidelines for Medical and Health Research Involving Human Subjects published by the Ministry of Health, Labor, and Welfare of Japan, and the use of anonymized data from the Japan Trauma Data Bank (JTDB), informed consent was waived. The approval document from the Japanese Association for the Surgery of Trauma and the representative institution (National Defense Medical College Research Institute) are available on the JTDB website (https://www.jtcr-jatec.org/traumabank/dataroom/ethics2.htm) (approval ID No. 2548).

### Meeting presentations

The results of this study were partially presented at the 19th European Congress of Trauma & Emergency Surgery in May 2018 at Valencia, Spain and the 32nd Annual Congress of The European Society of Intensive Care Medicine in September 2019 in Berlin, Germany.

### Supplementary Information

Supplementary Information.

## Data Availability

The datasets used and analyzed for this study are available from the corresponding author upon reasonable request.

## Abbreviations

SAE: Splenic artery embolization
BSI: Blunt splenic injury
RR: Relative risk
CI: Confidence interval
AIS: Abbreviated Injury Scale
JTDB: Japan Trauma Data Bank
STROBE: Strengthening the Reporting of Observational Studies in Epidemiology
IQR: Interquartile range
