# Supplementary material for: Comparative effectiveness of angioembolization versus open surgery in patients with blunt splenic injury
Source: Sci Rep. 2024 Apr 16;14:8800. doi: 10.1038/s41598-024-59420-w (PMC11021531; doi:10.1038/s41598-024-59420-w)
Supplement: Supplementary file 1 — Supplementary Information. [file 41598_2024_59420_MOESM1_ESM.docx]

**Supplementary material**

**Comparative effectiveness of angioembolization versus open surgery in patients with blunt splenic injury**

**Appendix E1.** Method of patient assignment to the SAE or open surgery group.

**Appendix E2.** Details regarding complications as secondary outcomes.

**Appendix E3.** Data preparation for statistical analysis.

**Appendix E4.** Variables for the generalized linear mixed model in the primary analysis.

**Appendix E5.** Four sensitivity analyses.

**Table E1.** Baseline characteristics before and after matching with cluster-exact (hospital and onset year) propensity score matching as the first sensitivity analysis.

**Table E2.** Resuscitative management before and after matching with cluster-exact (hospital and onset year) propensity score matching as the first sensitivity analysis.

**Table E3.** Baseline characteristics before and after matching without multiple imputation as the second sensitivity analysis.

**Table E4.** Resuscitative management before and after matching without multiple imputation as the second sensitivity analysis.

**Table E5.** Baseline characteristics primary analysis using AIS matching and sensitivity analysis using ISS matching.

**Table E6.** Resuscitative management primary analysis using AIS matching and sensitivity analysis using ISS matching.

**Table E7.** Study outcomes comparison between the primary analysis using AIS matching and the sensitivity analysis using ISS matching.

**Table E8.** The 92 variables from the Japan Trauma Data Bank that were used for this study.

**Table E9.** The AIS98 codes for splenic injury.

**Table E10.** The AIS98 codes for the study exclusion criteria.

**Table E11.** Missing data for the study participants (n = 2,192)

**Fig. E1.** Prevalence of splenic artery embolization in each hospital.

**Fig. E2.** Comparison of prevalence trends between splenic artery embolization and open surgery among patients with blunt splenic injury.

**Appendix E1. Method of patient assignment to the SAE or open surgery group**

Patients were assigned to the SAE or open surgery group based on which hemostasis treatment they received first. The JTDB includes codes for the following seven hemostasis-related procedures: total splenectomy, splenorrhaphy, crash laparotomy, partial splenectomy, abdominal transarterial embolization (TAE), emergency TAE, and TAE as a primary method for hemostasis. The first treatment that the patient received was identified from the code entered in the database. The SAE group included patients who had at least one code for SAE but did not have any for open surgeries. In contrast, the open surgery group included patients who had at least one code for open surgeries but did not have any for SAE. Patients who had codes for both open surgeries and SAE were considered to have undergone both SAE and open surgery sequentially. Among these patients, patients having a code for total splenectomy (SAE was usually omitted after total spleen resection) or who received TAE as the primary method for hemostasis were assigned to the SAE group. In contrast, patients who had a code for crash laparotomy were assigned to the open surgery group. Patients who could not be classified based on the above-mentioned rules were assigned to the SAE group because it is not common to perform SAE after open surgery.

**Appendix E2. Details regarding complications as secondary outcomes**

The classification of each complication (abdominal complications, organ failure-related complications, infectious complications, and central nervous system complications) was determined based on the codes for the complications recorded in the Japan Trauma Databank, including each of the following variables:

| Classifications | Variables recorded in the Japan Trauma Databank |
| --- | --- |
| Abdominal complications | Peptic ulcer  Ileus  Pancreatitis  Cholecystitis  Liver failure  Digestion-other  Abdominal abscess  Abdominal compartment syndrome |
| Organ failure-related complications | Acute renal failure  Lung edema  Acute respiratory distress syndrome  Disseminated intravascular coagulation  Thrombocytopenia  Coagulation-other  Sepsis and organ failure  Liver failure |
| Infectious complications | Pneumonia  Empyema  Bloodstream infection  Wound infection  Sepsis and organ failure  Urinary tract infection  Abdominal abscess |
| Central nervous system complications | Diabetes insipidus  Hydrocephalus  Fat embolism, meningitis  Neuropsychological deficit  Other complications in the brain |

**Appendix E3. Data preparation for statistical analysis**

The JTDB variables potentially contained outliers in numeric variables and contradictory values in pairs of the study variables, such as the negative value for the duration of the hospital stay. Negative values for the number of days of intensive care unit stay and hospital stay were considered to be contradictory and were transformed as missing data. A single-sample robust linear regression analysis with an M estimator was used to detect outliers for the following variables: age, systolic blood pressure, heart rate, body temperature, intensive care unit stay, and hospital stay. These variables were also transformed as missing data.

Multiple imputation by chained equation was conducted to impute missing values for the study variables (Table E11). This generated 25 multiply imputed datasets. Statistical analyses were performed on each imputed dataset, which were further integrated using the Rubin’s rule [1].

**Appendix E4. Variables for the generalized linear mixed model in the primary analysis**

In the primary analysis, the propensity scores for undergoing splenic artery embolization were estimated using a logistic-regression mixed effect model with 34 fixed-effect explanatory variables and two random-effect explanatory variables. The 34 fixed-effect explanatory variables were age; sex; systolic blood pressure; heart rate; Glasgow Coma Scale score; body temperature; procedures undergone during the stay in the trauma bay including airway management, chest drain, vasopressor, aortic occlusion, and blood transfusion; the maximum Abbreviated Injury Scale (for body regions 1–9); preexisting comorbidities including coronary heart disease, congestive heart failure, hypertension, stroke, dementia, chronic obstructive pulmonary disease, peptic ulcer, liver cirrhosis, diabetes mellitus, chronic renal failure, solid malignancy, hematological disease, and acquired immunodeficiency syndrome; and AIS codes of splenic injury. The two random-effect explanatory variables were the institution identifier and period of onset year (2004–2011 versus 2012–2019). In addition, to achieve appropriate balancing between the matched groups, two interaction terms (airway management: blood transfusion and blood transfusion: Glasgow Coma Scale score) were included in the regression model for the propensity score estimation.

**Appendix E5. Four sensitivity analyses**

Four sensitivity analyses were performed to test the robustness of the primary analyses. First, to account for disparities between facilities, cluster-exact propensity score matching was used to select 1:1 matched pairs from the same institution identifier (e.g., same hospital) and the same period of injury. Second, propensity score matching was used for the naïve dataset without multiple imputations to test the robustness of the multiple imputations. Third, a generalized linear mixed effects model was used with the imputed data without matching to test the robustness of the propensity score-based matching. Fourth, to use a simpler severity score, the Injury Severity Score was used for matching instead of the AIS score, and the analysis method itself was the same as the primary analysis. Outcome measures of the first two sensitivity analyses were assessed using the double-adjustment method similar to the primary analyses. Propensity scores for undergoing SAE were estimated using a generalized linear mixed effect model with the same explanatory variables, several interaction terms, and two random effects (institution identifier and period).

In the sensitivity analyses, propensity scores for undergoing splenic artery embolization were estimated using a generalized linear mixed model with the explanatory variables, interaction terms, and two random effects (institution identifier and period) similar to those in the primary analysis (Appendix E4).

The explanatory variables used in the sensitivity analysis were the same as those in the primary analysis.

In the first sensitivity analysis, the five interaction terms were dementia: maximum AIS for body region 1, dementia: maximum AIS for body region 2, dementia: Glasgow Coma Scale score, and stroke: aortic occlusion.

In the second sensitivity analysis, the single interaction term was airway management: chest drain.

In the fourth sensitivity analysis, the three interaction terms were airway management: Blood transfusion, airway management: systolic blood pressure, and Chest drain: systolic blood pressure.

**Table E1. Baseline characteristics before and after matching in the first sensitivity analysis.**

|  | **Before Matching** | | |  | **After Matching Without MI** | | |
| --- | --- | --- | --- | --- | --- | --- | --- |
| **Variables** | **SAE** | **Open surgery** | **SMD** |  | **SAE** | **Open surgery** | **SMD** |
| No. of subjects | 1,634 | 558 |  |  | 220 | 220 |  |
| Sex, Female, n (%) | 470 (28.8) | 123 (22.0) | 0.155 |  | 55 (25.1) | 48 (21.8) | 0.078 |
| Age, in years, (SD) | 42 (23) | 42 (22) | 0.033 |  | 41 (22) | 41 (22) | 0.001 |
| Vital signs at hospital arrival |  |  |  |  |  |  |  |
| Systolic blood pressure, mmHg, (SD) | 114 (29) | 102 (32) | 0.380 |  | 106 (29) | 106 (31) | 0.01 |
| Heart rate, beats per minute, (SD) | 95 (25) | 99 (27) | 0.160 |  | 100 (27) | 98 (25) | 0.055 |
| Body temperature, °C, (SD) | 36.3 (0.9) | 36.0 (1.1) | 0.297 |  | 36.1 (1.0) | 36.1 (1.0) | 0.044 |
| Glasgow Coma Scale, median [IQR] | 15 [14, 15] | 14 [12, 15] | 0.298 |  | 14 [12, 15] | 14 [12, 15] | 0.050 |
| Medical history |  |  |  |  |  |  |  |
| Coronary heart disease | 33 (2.0) | 9 (1.6) | 0.030 |  | 5 (2.2) | 3 (1.5) | 0.049 |
| Congestive heart failure | 15 (0.9) | 6 (1.1) | 0.016 |  | 3 (1.4) | 2 (0.9) | 0.043 |
| Hypertension | 171 (10.5) | 54 (9.7) | 0.026 |  | 22 (10.2) | 21 (9.5) | 0.025 |
| Stroke | 33 (2.0) | 11 (2.0) | 0.003 |  | 1 (0.4) | 2 (0.9) | 0.065 |
| Dementia | 20 (1.2) | 9 (1.6) | 0.033 |  | 2 (0.8) | 0 (0.0) | 0.128 |
| Chronic obstructive pulmonary disease | 12 (0.7) | 1 (0.2) | 0.082 |  | 0 (0.0) | 0 (0.0) | 0.027 |
| Peptic ulcer | 13 (0.8) | 6 (1.1) | 0.029 |  | 1 (0.6) | 2 (0.9) | 0.033 |
| Liver cirrhosis | 15 (0.9) | 10 (1.8) | 0.076 |  | 2 (0.9) | 4 (1.7) | 0.071 |
| Diabetes mellitus | 89 (5.4) | 34 (6.1) | 0.028 |  | 16 (7.2) | 11 (5.1) | 0.090 |
| Chronic renal failure | 19 (1.2) | 3 (0.5) | 0.068 |  | 1 (0.5) | 1 (0.5) | <0.001 |
| Malignancy | 16 (1.0) | 6 (1.1) | 0.010 |  | 1 (0.3) | 1 (0.5) | 0.027 |
| Hematological disease | 2 (0.1) | 3 (0.5) | 0.072 |  | 0 (0.0) | 2 (0.9) | 0.127 |
| Human immunodeficiency virus | 1 (0.1) | 0 (0.0) | 0.035 |  | 0 (0.0) | 0 (0.0) | <0.001 |
| AIS code for splenic injury, n (%) |  |  |  |  |  |  |  |
| NFS(544299.2) | 68 (4.2) | 18 (3.2) | 0.05 |  | 12 (5.3) | 10 (4.5) | 0.034 |
| **Contusion** |  |  |  |  |  |  |  |
| NFS (544210.2) | 44 (2.7) | 7 (1.3) | 0.104 |  | 2 (0.9) | 6 (2.7) | 0.138 |
| Minor, superficial; OIS I, II (544212.2) | 172 (10.5) | 28 (5.0) | 0.207 |  | 14 (6.5) | 20 (9.1) | 0.097 |
| Major; OIS III (544214.3) | 174 (10.6) | 30 (5.4) | 0.195 |  | 14 (6.3) | 16 (7.2) | 0.036 |
| **Laceration** |  |  |  |  |  |  |  |
| NFS (544220.2) | 23 (1.4) | 9 (1.6) | 0.017 |  | 7 (3.0) | 2 (1.1) | 0.135 |
| Minor, superficial; OIS I, II (544222.2) | 98 (6.0) | 37 (6.6) | 0.026 |  | 24 (11.1) | 20 (9.0) | 0.071 |
| Moderate; OIS III (544224.3) | 630 (38.6) | 136 (24.4) | 0.309 |  | 65 (29.7) | 61 (27.6) | 0.048 |
| Major; OIS IV (544226.4) | 352 (21.5) | 198 (35.5) | 0.313 |  | 62 (28.2) | 73 (33.4) | 0.113 |
| Massive; OIS V (544228.5) | 42 (2.6) | 63 (11.3) | 0.349 |  | 12 (5.4) | 7 (3.1) | 0.114 |
| **Rupture** |  |  |  |  |  |  |  |
| NFS (544240.3) | 43 (2.6) | 35 (6.3) | 0.177 |  | 11 (4.8) | 6 (2.9) | 0.099 |
| **Injury Severity Score**^*^,**median [IQR]** | 22 [13–34] | 25 [16–36] | 0.246 |  | 25 [16–36] | 25 [16–36] | 0.065 |
| AIS body region, median score [IQR] |  |  |  |  |  |  |  |
| AIS 1: Head | 0 [0, 2] | 0 [0, 1] | 0.075 |  | 0 [0, 3] | 0 [0, 2] | 0.056 |
| AIS 2: Face | 0 [0, 0] | 0 [0, 0] | 0.008 |  | 0 [0, 0] | 0 [0, 0] | 0.004 |
| AIS 3: Neck | 0 [0, 0] | 0 [0, 0] | 0.090 |  | 0 [0, 0] | 0 [0, 0] | 0.068 |
| AIS 4: Thorax | 3 [0, 4] | 3 [0, 4] | 0.091 |  | 3 [0, 4] | 3 [0, 4] | 0.025 |
| AIS 5: Abdomen^*^ | 3 [3, 4] | 3 [3, 4] | 0.467 |  | 3 [3, 4] | 3 [3, 4] | 0.011 |
| AIS 6: Spine | 0 [0, 0] | 0 [0, 0] | 0.030 |  | 0 [0, 0] | 0 [0, 0] | 0.023 |
| AIS 7: Upper extremity | 0 [0, 1] | 0 [0, 1] | 0.064 |  | 0 [0, 2] | 0 [0, 1] | 0.099 |
| AIS 8: Lower extremity | 0 [0, 2] | 0 [0, 2] | 0.089 |  | 0 [0, 2] | 0 [0, 2] | 0.009 |
| AIS 9: Unspecified | 0 [0, 0] | 0 [0, 0] | 0.046 |  | 0 [0, 0] | 0 [0, 0] | 0.056 |
| **Onset year** |  |  |  |  |  |  |  |
| 2004–2011 | 510 (31.4) | 256 (46.0) | 0.30 |  | 84 (38.3) | 84 (38.3) | <0.001 |
| 2012–2019 | 1,115 (68.6) | 301 (54.0) | - |  | 136 (61.7) | 136 (61.7) | — |
| NA | 9 | 1 |  |  |  |  |  |

The variables listed in the table were used to estimate the propensity score in the first sensitivity analysis to predict the possibility of undergoing SAE.
*, Not included in variables to estimate propensity score.

Categorical and continuous variables are expressed as absolute counts (%) and mean (SD), respectively, unless otherwise specified. The Glasgow Coma Scale score and AIS body region are expressed as median [IQR].

Abbreviations: –, not available; AIS, Abbreviated Injury Scale; IQR, interquartile range; MI, multiple imputation; NFS, not further specified; OIS, Organ Injury Scale; SAE, splenic artery embolization; SMD, standardized mean difference.

**Table E2. Resuscitative management before and after matching in the first sensitivity analysis**

|  | **Before Matching** | | |  | **After Matching Without MI** | | |
| --- | --- | --- | --- | --- | --- | --- | --- |
| Procedure performed in the trauma bay | SAE  N=1,634 | Open surgery  N=558 | SMD |  | SAE  N=220 | Open surgery  N=220 | SMD |
| Airway management | 512 (31.3) | 275 (49.3) | 0.372 |  | 101 (45.7) | 104 (47.2) | 0.030 |
| Chest drain | 293 (17.9) | 146 (26.2) | 0.200 |  | 54 (24.4) | 57 (25.8) | 0.031 |
| Vasopressor administration | 88 (5.4) | 67 (12.0) | 0.237 |  | 17 (7.8) | 22 (9.8) | 0.072 |
| Aortic occlusion | 46 (2.8) | 50 (9.0) | 0.263 |  | 8 (3.8) | 13 (5.7) | 0.091 |
| Blood transfusion | 614 (37.6) | 314 (56.3) | 0.381 |  | 102 (46.2) | 105 (47.8) | 0.033 |

All variables listed in the table were used to estimate the propensity score in the first sensitivity analysis to predict the possibility of undergoing SAE.

Resuscitation management is generally considered to be an important step that is taken in the trauma bay to correct the physiological disorders of trauma patients. Variables for resuscitation management were selected by the authors for inclusion in the model. Categorical variables are expressed as absolute counts (%).

Abbreviations: SAE, splenic artery embolization; SMD, standardized mean difference.

**Table E3. Baseline characteristics before and after matching in the second sensitivity analysis**

|  | **Before Matching** | | |  | **After Matching Without MI** | | |
| --- | --- | --- | --- | --- | --- | --- | --- |
| **Variables** | **SAE** | **Open surgery** | **SMD** |  | **SAE** | **Open surgery** | **SMD** |
| No. of subjects | 1,634 | 558 |  |  | 259 | 259 |  |
| Sex, female, n (%) | 470 (28.8) | 123 (22.0) | 0.155 |  | 64 (24.7) | 68 (26.3) | 0.035 |
| Age, in years, (SD) | 42 (23) | 42 (22) | 0.033 |  | 43 (23) | 42 (21) | 0.060 |
| Vital signs at hospital arrival |  |  |  |  |  |  |  |
| Systolic blood pressure, mmHg, (SD) | 114 (29) | 102 (32) | 0.380 |  | 104 (29) | 107 (29) | 0.105 |
| Heart rate, beats per minute, (SD) | 95 (25) | 99 (27) | 0.160 |  | 98 (26) | 96 (24) | 0.081 |
| Body temperature, °C, (SD) | 36.3 (0.9) | 36.0 (1.1) | 0.297 |  | 36.1 (1.0) | 36.1 (1.0) | 0.81 |
| Glasgow Coma Scale, median [IQR] | 15 [14, 15] | 14 [12, 15] | 0.298 |  | 14 [11, 15] | 15 [13, 15] | 0.192 |
| Medical history, n (%) |  |  |  |  |  |  |  |
| Coronary heart disease | 33 (2.0) | 9 (1.6) | 0.030 |  | 5 (1.9) | 4 (1.5) | 0.030 |
| Congestive heart failure | 15 (0.9) | 6 (1.1) | 0.016 |  | 5 (1.9) | 2 (0.8) | 0.100 |
| Hypertension | 171 (10.5) | 54 (9.7) | 0.026 |  | 28 (10.8) | 24 (9.3) | 0.051 |
| Stroke | 33 (2.0) | 11 (2.0) | 0.003 |  | 6 (2.3) | 4 (1.5) | 0.056 |
| Dementia | 20 (1.2) | 9 (1.6) | 0.033 |  | 6 (2.3) | 3 (1.2) | 0.089 |
| Chronic obstructive pulmonary disease | 12 (0.7) | 1 (0.2) | 0.082 |  | 1 (0.4) | 1 (0.4) | <0.001 |
| Peptic ulcer | 13 (0.8) | 6 (1.1) | 0.029 |  | 1 (0.4) | 3 (1.2) | 0.088 |
| Liver cirrhosis | 15 (0.9) | 10 (1.8) | 0.076 |  | 4 (1.5) | 4 (1.5) | <0.001 |
| Diabetes mellitus | 89 (5.4) | 34 (6.1) | 0.028 |  | 17 (6.6) | 16 (6.2) | 0.016 |
| Chronic renal failure | 19 (1.2) | 3 (0.5) | 0.068 |  | 2 (0.8) | 1 (0.4) | 0.051 |
| Malignancy | 16 (1.0) | 6 (1.1) | 0.010 |  | 3 (1.2) | 1 (0.4) | 0.088 |
| Hematological disease | 2 (0.1) | 3 (0.5) | 0.072 |  | 2 (0.8) | 1 (0.4) | 0.051 |
| Human immunodeficiency virus | 1 (0.1) | 0 (0.0) | 0.035 |  | 0 (0.0) | 0 (0.0) | <0.001 |
| AIS code for splenic injury |  |  |  |  |  |  |  |
| NFS(544299.2) | 68 (4.2) | 18 (3.2) | 0.05 |  | 3 (1.2) | 6 (2.3) | 0.089 |
| **Contusion** |  |  |  |  |  |  |  |
| NFS (544210.2) | 44 (2.7) | 7 (1.3) | 0.104 |  | 5 (1.9) | 5 (1.9) | <0.001 |
| Minor, superficial; OIS I, II (544212.2) | 172 (10.5) | 28 (5.0) | 0.207 |  | 18 (6.9) | 19 (7.3) | 0.015 |
| Major; OIS III (544214.3) | 174 (10.6) | 30 (5.4) | 0.195 |  | 13 (5.0) | 21 (8.1) | 0.125 |
| **Laceration** |  |  |  |  |  |  |  |
| NFS (544220.2) | 23 (1.4) | 9 (1.6) | 0.017 |  | 5 (1.9) | 4 (1.5) | 0.03 |
| Minor, superficial; OIS I, II (544222.2) | 98 (6.0) | 37 (6.6) | 0.026 |  | 24 (9.3) | 23 (8.9) | 0.013 |
| Moderate; OIS III (544224.3) | 630 (38.6) | 136 (24.4) | 0.309 |  | 63 (24.3) | 82 (31.7) | 0.164 |
| Major; OIS IV (544226.4) | 352 (21.5) | 198 (35.5) | 0.313 |  | 101 (39.0) | 80 (30.9) | 0.171 |
| Massive; OIS V (544228.5) | 42 (2.6) | 63 (11.3) | 0.349 |  | 19 (7.3) | 11 (4.2) | 0.133 |
| **Rupture** |  |  |  |  |  |  |  |
| NFS (544240.3) | 43 (2.6) | 35 (6.3) | 0.177 |  | 11 (4.2) | 11 (4.2) | <0.001 |
| **Injury Severity Score^*^, median [IQR]** | 22 [13–34] | 25 [16–36] | 0.246 |  | 29 [18–41] | 25 [16–34] | 0.316 |
| AIS body region, score, median [IQR] |  |  |  |  |  |  |  |
| AIS 1: Head | 0 [0, 2] | 0 [0, 1] | 0.075 |  | 0 [0, 3] | 0 [0, 1] | 0.161 |
| AIS 2: Face | 0 [0, 0] | 0 [0, 0] | 0.008 |  | 0 [0, 0] | 0 [0, 0] | 0.006 |
| AIS 3: Neck | 0 [0, 0] | 0 [0, 0] | 0.090 |  | 0 [0, 0] | 0 [0, 0] | 0.125 |
| AIS 4: Thorax | 3 [0, 4] | 3 [0, 4] | 0.091 |  | 3 [0, 4] | 3 [0, 4] | 0.179 |
| AIS 5: Abdomen^*^ | 3 [3, 4] | 3 [3, 4] | 0.467 |  | 3 [3, 4] | 3 [3, 4] | 0.165 |
| AIS 6: Spine | 0 [0, 0] | 0 [0, 0] | 0.030 |  | 0 [0, 0] | 0 [0, 0] | 0.074 |
| AIS 7: Upper extremity | 0 [0, 1] | 0 [0, 1] | 0.064 |  | 0 [0, 2] | 0 [0, 1] | 0.032 |
| AIS 8: Lower extremity | 0 [0, 2] | 0 [0, 2] | 0.089 |  | 0 [0, 2] | 0 [0, 2] | 0.082 |
| AIS 9: Unspecified | 0 [0, 0] | 0 [0, 0] | 0.046 |  | 0 [0, 0] | 0 [0, 0] | 0.076 |

*, Not included in variables to estimate propensity score.

Categorical and continuous variables are expressed as absolute counts (%) and mean (SD), respectively, unless otherwise specified. The Glasgow Coma Scale score and AIS body region are expressed as median [IQR].

Abbreviations: –, not available; AIS, Abbreviated Injury Scale; IQR, interquartile range; MI, multiple imputation; NFS, not further specified; OIS, Organ Injury Scale; SAE, splenic artery embolization; SD, standard deviation; SMD, standardized mean difference.

**Table E4. Resuscitative management before and after matching in the second sensitivity analysis**

|  | **Before Matching** | | |  | **After Matching Without MI** | | |
| --- | --- | --- | --- | --- | --- | --- | --- |
| Procedures performed in the trauma bay | SAE  N=1,634 | Open surgery  N=558 | SMD |  | SAE  N=259 | Open surgery  N=259 | SMD |
| Airway management | 512 (31.3) | 275 (49.3) | 0.372 |  | 140 (54.1) | 114 (44.0) | 0.202 |
| Chest drain | 293 (17.9) | 146 (26.2) | 0.200 |  | 80 (30.9) | 69 (26.6) | 0.094 |
| Vasopressor administration | 88 (5.4) | 67 (12.0) | 0.237 |  | 35 (13.5) | 29 (11.2) | 0.070 |
| Aortic occlusion | 46 (2.8) | 50 (9.0) | 0.263 |  | 22 (8.5) | 14 (5.4) | 0.122 |
| Blood transfusion | 614 (37.6) | 314 (56.3) | 0.381 |  | 146 (56.4) | 128 (49.4) | 0.140 |

All the variables listed in the table were used to estimate the propensity score to predict the possibility of undergoing SAE.

Resuscitation management is an important step that must be taken in the trauma bay, if needed, to correct physiological disorders. Categorical variables are expressed as absolute counts (%).

Abbreviations: SAE, splenic artery embolization; SMD, standardized mean difference.

**Table E5.** Baseline characteristics primary analysis using AIS matching and sensitivity analysis using ISS matching

|  | **Primary Analysis using AIS matching** | | |  | | **Sensitivity Analysis using ISS matching** | | | |  |
| --- | --- | --- | --- | --- | --- | --- | --- | --- | --- | --- |
|  | **SAE** | **Open surgery** | **SMD** | |  | | **SAE** | **Open surgery** | **SMD** | |
| **Patients** | 377 | 377 |  | |  | | 385 | 385 |  | |
| **Sex, female, n (%)** | 80 (21.3) | 93 (24.8) | 0.081 | |  | | 84 (21.8) | 96 (24.9) | 0.072 | |
| **Age, in years, (SD)** | 43 (23) | 42 (22) | 0.040 | |  | | 43 (23) | 42 (22) | 0.059 | |
| **Vital signs at hospital arrival** |  |  |  | |  | |  |  |  | |
| Systolic blood pressure, mmHg, (SD) | 102 (30) | 105 (32) | 0.074 | |  | | 102 (30) | 104 (31) | 0.055 | |
| Heart rate, beats/min, (SD) | 100 (26) | 98 (26) | 0.066 | |  | | 99 (26) | 99 (26) | 0.032 | |
| Body temperature, °C, (SD) | 36.0 (1.1) | 36.1 (1.0) | 0.061 | |  | | 36.0 (1.1) | 36.1 (1.0) | 0.057 | |
| GCS, median [IQR] | 14 [12–15] | 14 [13–15] | 0.110 | |  | | 14 [12–15] | 14 [12–15] | 0.088 | |
| **Medical history, n (%)** |  |  |  | |  | |  |  |  | |
| Coronary heart disease | 7 (1.8) | 6 (1.6) | 0.019 | |  | | 8 (2.0) | 6 (1.6) | 0.030 | |
| Congestive heart failure | 5 (1.2) | 3 (0.8) | 0.044 | |  | | 5 (1.2) | 3 (0.8) | 0.043 | |
| Hypertension | 33 (8.7) | 34 (8.9) | 0.01 | |  | | 35 (9.0) | 33 (8.5) | 0.017 | |
| Stroke | 7 (1.8) | 5 (1.3) | 0.042 | |  | | 8 (2.1) | 5 (1.3) | 0.060 | |
| Dementia | 7 (1.9) | 5 (1.3) | 0.046 | |  | | 7 (1.9) | 5 (1.4) | 0.037 | |
| Chronic obstructive pulmonary disease | 2 (0.4) | 1 (0.3) | 0.017 | |  | | 1 (0.3) | 1 (0.3) | 0.010 | |
| Peptic ulcer | 3 (0.9) | 4 (1.1) | 0.02 | |  | | 3 (0.8) | 4 (1.0) | 0.030 | |
| Liver cirrhosis | 6 (1.6) | 4 (1.1) | 0.046 | |  | | 6 (1.5) | 5 (1.2) | 0.024 | |
| Diabetes mellitus | 25 (6.6) | 23 (6.0) | 0.022 | |  | | 25 (6.5) | 23 (6.1) | 0.016 | |
| Chronic renal failure | 1 (0.3) | 2 (0.5) | 0.044 | |  | | 1 (0.3) | 2 (0.5) | 0.042 | |
| Malignancy | 2 (0.6) | 2 (0.5) | 0.011 | |  | | 3 (0.7) | 2 (0.5) | 0.026 | |
| Hematological disease | 2 (0.5) | 1 (0.3) | 0.042 | |  | | 2 (0.4) | 1 (0.3) | 0.022 | |
| Human immunodeficiency virus | 0 (0.0) | 0 (0.0) | <0.001 | |  | | 0 (0.0) | 0 (0.0) | <0.001 | |
| **AIS code for splenic injury, n (%)** |  |  |  | |  | |  |  |  | |
| NFS(544299.2) | 16 (4.3) | 15 (4.1) | 0.011 | |  | | 16 (4.1) | 13 (3.5) | 0.031 | |
| **Contusion** |  |  |  | |  | |  |  |  | |
| NFS (544210.2) | 5 (1.4) | 6 (1.6) | 0.018 | |  | | 5 (1.4) | 6 (1.5) | 0.010 | |
| Minor, superficial; OIS I, II (544212.2) | 21 (5.5) | 25 (6.6) | 0.048 | |  | | 22 (5.6) | 25 (6.5) | 0.036 | |
| Major; OIS III (544214.3) | 25 (6.5) | 29 (7.6) | 0.043 | |  | | 22 (5.8) | 28 (7.3) | 0.061 | |
| **Laceration** |  |  |  | |  | |  |  |  | |
| NFS (544220.2) | 7 (1.8) | 6 (1.6) | 0.016 | |  | | 7 (1.9) | 6 (1.6) | 0.024 | |
| Minor, superficial; OIS I, II (544222.2) | 29 (7.8) | 29 (7.7) | 0.005 | |  | | 28 (7.4) | 28 (7.3) | 0.004 | |
| Moderate; OIS III (544224.3) | 92 (24.3) | 113 (29.9) | 0.125 | |  | | 92 (23.8) | 114 (29.6) | 0.133 | |
| Major; OIS IV (544226.4) | 140 (37.1) | 124 (32.9) | 0.087 | |  | | 144 (37.4) | 130 (33.7) | 0.076 | |
| Massive; OIS V (544228.5) | 25 (6.7) | 18 (4.7) | 0.089 | |  | | 29 (7.6) | 21 (5.5) | 0.084 | |
| **Rupture** |  |  |  | |  | |  |  |  | |
| NFS (544240.3) | 20 (5.3) | 15 (4.1) | 0.056 | |  | | 22 (5.7) | 17 (4.3) | 0.067 | |
| **Injury Severity Score^*^ ,median [IQR]** | 27 [17–36] | 25 [16–36] | 0.130 | |  | | 27 [17–36] | 26 [17–36] | 0.051 | |
| **AIS body region, median score [IQR]** |  |  |  | |  | |  |  |  | |
| AIS 1: Head | 0 [0–2] | 0 [0–1] | 0.032 | |  | | 0 [0–2] | 0 [0–2] | 0.025 | |
| AIS 2: Face | 0 [0–0] | 0 [0–0] | 0.013 | |  | | 0 [0–0] | 0 [0–0] | 0.024 | |
| AIS 3: Neck | 0 [0–0] | 0 [0–0] | 0.001 | |  | | 0 [0–0] | 0 [0–0] | 0.136 | |
| AIS 4: Thorax | 3 [0–4] | 3 [0–4] | 0.041 | |  | | 3 [0–4] | 3 [0–4] | 0.066 | |
| AIS 5: Abdomen^**^ | 3 [3–4] | 3 [3–4] | 0.094 | |  | | 3 [3–4] | 3 [3–4] | 0.078 | |
| AIS 6: Spine | 0 [0–0] | 0 [0–0] | 0.025 | |  | | 0 [0–0] | 0 [0–0] | 0.076 | |
| AIS 7: Upper extremity | 0 [0–1] | 0 [0–1] | 0.02 | |  | | 0 [0–1] | 0 [0–1] | 0.006 | |
| AIS 8: Lower extremity | 0 [0–2] | 0 [0–2] | 0.005 | |  | | 0 [0–2] | 0 [0–2] | 0.154 | |
| AIS 9: Unspecified | 0 [0–0] | 0 [0–0] | 0.027 | |  | | 0 [0–0] | 0 [0–0] | 0.091 | |

The variables listed in the table were used to estimate the propensity score to predict the likelihood of undergoing SAE.

*, Not included in variables to estimate propensity score in Primary Analysis using AIS matching.
**, Not included in variables to estimate propensity score.

Categorical and continuous variables are expressed as absolute counts (%) and mean (SD), respectively, unless otherwise specified. GCS and AIS body region are expressed as median IQR.

Abbreviations: AIS, Abbreviated Injury Scale; GCS, Glasgow Coma Scale; ISS, Injury Severity Score; IQR, interquartile range; NFS, not further specified; OIS, Organ Injury Scale; SD, standard deviation; SMD, standardized mean difference

**Table E6.** Resuscitative management primary analysis using AIS matching and sensitivity analysis using ISS matching

|  | **Primary Analysis using AIS matching** | | |  | **Sensitivity Analysis using ISS matching** | | |
| --- | --- | --- | --- | --- | --- | --- | --- |
| **Procedures performed in the trauma bay** | **SAE**  **N=379** | **Open surgery**  **N=379** | **SMD** |  | **SAE**  **N=385** | **Open surgery**  **N=385** | **SMD** |
| Airway management, n (%) | 194 (51.2) | 169 (44.7) | 0.129 |  | 200 (51.9) | 170 (44.1) | 0.156 |
| Chest drain | 107 (28.4) | 99 (26.1) | 0.05 |  | 109 (28.2) | 99 (25.7) | 0.056 |
| Vasopressor administration | 45 (12.0) | 38 (10.1) | 0.059 |  | 46 (12.0) | 43 (11.2) | 0.027 |
| Aortic occlusion | 30 (8.0) | 21 (5.5) | 0.099 |  | 29 (7.6) | 23 (5.9) | 0.070 |
| Blood transfusion | 213 (56.2) | 192 (50.7) | 0.110 |  | 216 (56.2) | 196 (50.9) | 0.108 |

All variables listed in the table were used to estimate the propensity score to predict the likelihood of undergoing SAE.

Resuscitation management is an important step needed to correct physiological disorders that must be performed in trauma bays. Categorical variables are expressed as absolute counts (%).

Abbreviations: AIS, Abbreviated Injury Scale; ISS, Injury Severity Score; SAE, splenic artery embolization; SMD, standardized mean difference

**Table E7.** Study outcomes comparison between primary analysis using AIS matching and sensitivity analysis using ISS matching

| **Primary Analysis using AIS matching** | | | | | |
| --- | --- | --- | --- | --- | --- |
| **Outcomes** | **SAE**  **N=377** | **Open surgery**  **N=377** | **Relative risk or difference** | | ***P* value** |
|  |  |  | Unadjusted | Adjusted |  |
| In-hospital mortality, n (%) | 44 (11.6%) | 42 (11.2%) | 1.03 [0.67–1.59] | 0.64 [0.38–1.09] | 0.10 |
| Spleen salvage, n (%) | 327 (87.1%) | 121 (32.1%) | 2.71 [2.37–3.10] | 2.84 [2.29–3.51] | < 0.001 |
| Hospital-free days at day 28, median [IQR] | 1 [0, 13] | 2 [0, 14] | 0 [−1 to 1] | 0 [−1 to 1] | 0.74 |
| **Sensitivity Analysis using ISS matching** | | | | | |
| **Outcomes** | **SAE**  **N=385** | **Open surgery**  **N=385** | **Relative risk or difference** | | ***P* value** |
|  |  |  | Unadjusted | Adjusted |  |
| In-hospital mortality, n (%) | 44 (11.4%) | 45 (11.6%) | 0.98 [0.65–1.48] | 0.62 [0.37–1.02] | 0.06 |
| Spleen salvage, n (%) | 331 (86.1%) | 118 (30.6%) | 2.81 [2.44–3.24] | 2.95 [2.37–3.67] | < 0.001 |
| Hospital-free days at day 28, median [IQR] | 1 [0, 13] | 1 [0, 14] | 0 [−1 to 1] | 0 [−1 to 1] | 0.65 |

Spleen salvage was defined as the avoidance of total splenectomy.
The number of hospital-free days at day 28 was defined as the number of days that the patient was alive and not hospitalized during the first 28 days after hospital admission. The definition of complications is described in Appendix E1. Data are reported as number (%) or relative risk/difference [95% confidence interval]

Abbreviations: AIS, Abbreviated Injury Scale; CNS, central nervous system; IQR, interquartile range; ISS, Injury Severity Score; SAE, splenic artery embolization

**Table E8. Variables included in the Japan Trauma Data Bank that were used for this study**

| Age |
| --- |
| Sex |
| Vital signs on arrival at the emergency department (e.g., systolic blood pressure, heart rate, and body temperature) |
| Glasgow Coma Scale |
| Preexisting comorbidities (coronary heart disease, congestive heart failure, hypertension, stroke, dementia, chronic obstructive pulmonary disease, peptic ulcer, liver cirrhosis, diabetes mellitus, solid malignancy, hematological disease, and adult immunodeficiency syndrome) |
| AIS scores indicating splenic injury (AIS codes 544299, 544210, 544212, 544214, 544220, 544222, 544224, 544226, 544228, and 544240) |
| Injury Severity Score |
| Maximum AIS score (9 variables) |
| Procedures undergone during the stay in the trauma bay (oral intubation, nasal intubation, cricothyroidotomy, ventilator, chest drain, vasopressor administration, closed-chest or open-chest cardiopulmonary resuscitation, aortic cross clamping, and resuscitative endovascular balloon occlusion of the aorta) |
| Complications that occurred during hospitalization (peptic ulcer, ileus, pancreatitis, cholecystitis, liver failure, digestion-other, abdominal abscess, abdominal compartment syndrome, acute myocardial infarction, critical arrhythmia, acute renal failure, lung edema, acute respiratory distress syndrome, disseminated intravascular coagulation, thrombocytopenia, other coagulation disorders, pneumoniae, empyema, bloodstream infection, wound infection, sepsis and organ failure, urinary tract infection, diabetes insipidus, hydrocephalus, fat embolism, meningitis, neuropsychological deficit, and other neurological disorders) |
| Outcome (death or survival) |
| Discharge destination |
| Length of ICU stay and hospital stay |
| Institutional identifier |
| Year of suffering the injury |
| Hemostasis techniques (total splenectomy, partial splenectomy, splenorrhaphy, emergency laparotomy, abdominal TAE, emergency TAE, and TAE) |
| Pre-hospital cardiopulmonary resuscitation performance |
| Blood transfusion within 24 h |
| Trauma mechanism (blunt, penetrating, burn, and others) |

Abbreviations: AIS, Abbreviated Injury Scale; ICU, intensive care unit; TAE, transarterial embolization.

**Table E9. The AIS98 codes used for the study inclusion criteria**

| **AIS 98 code** | **AIS score** | **Specifications** |
| --- | --- | --- |
| 544299 | 2 | Not further specified |
| 544210 | 2 | Contusion (hematoma), not further specified |
| 544212 | 2 | Subcapsular, ≤50% surface area; intraparenchymal, nonexpanding, <5 cm in diameter; minor; superficial (OIS Grade Ⅰ or Ⅱ) |
| 544214 | 3 | Subcapsular, >50% surface area or expanding; ruptured subcapsular or parenchymal; intraparenchymal >5 cm in diameter or expanding; major (OIS Grade Ⅲ) |
| 544220 | 2 | Laceration, not further specified |
| 544222 | 2 | Simple capsular tear ≤3 cm in parenchymal depth; no major (i.e., trabecular) vessel involvement; minor; superficial (OIS Grade Ⅰ or Ⅱ) |
| 544224 | 3 | No hilar or segmental parenchymal disruption or destruction; >3 cm parenchymal depth or involving major (i.e., trabecular) vessels; moderate (OIS Grade Ⅲ) |
| 544226 | 4 | Involvement of segmental or hilar vessels producing major devascularization for >2% of the spleen but no hilar injury; major (OIS Grade Ⅳ) |
| 544228 | 5 | Hilar disruption producing total devascularization: tissue loss; avulsion; stellate; massive (OIS Grade Ⅴ) |
| 544240 | 3 | Rupture (fracture), not further specified |

Patients who had any of the above-mentioned codes were considered to have splenic injury and were included in this study.

Abbreviations: AIS, Abbreviated Injury Scale; OIS, Organ Injury Scale.

**Table E10. The AIS98 codes used for the study exclusion criteria**

| **Injury description (Abbreviated Injury Scale)** |
| --- |
| Adrenal gland (540226.3) |
| Anus (540424.3, 540426.4) |
| Bladder (540622.3, 540624.4, 540626.4, 540640.3) |
| Colon (540824.3, 540826.4) |
| Duodenum (541023.3, 541024.4, 541028.5) |
| Gallbladder (541224.3, 541226.4) |
| Jejunum-ileum (541424.3, 541426.4) |
| Kidney (541614.3, 541624.3, 541626.4, 541628.5, 541640.4) |
| Liver (541814.3, 541824.3, 541826.4, 541828.5, 541830.6, 541840.4) |
| Mesentery (542024.3, 542026.4) |
| Omentum (542224.3) |
| Pancreas (542814.3, 542824.3, 542826.4, 542828.4, 542830.4, 542832.5) |
| Rectum (543624.3, 543625.4, 543626.5) |
| Stomach (544424.3, 544426.4) |
| Abdominal aorta (520299.4, 520202.4, 520204.4, 520206.4, 520208.5) |
| Inferior vena cava (521299.3, 521202.3, 521204.3, 521206.4) |
| Diaphragm (440604.3, 440606.4) |

Patients who had any of these above-mentioned injuries were considered to have an abdominal organ injury outside the spleen and were excluded from the study.

**Table E11. Missing data for the study participants (n=2 192)**

|  | Missing data, n (%) |
| --- | --- |
| **Age** | 1 (0.04) |
| **Vital signs at hospital arrival** |  |
| Heart rate | 27 (1.2) |
| Systolic blood pressure | 24 (1.1) |
| Body temperature | 301 (13.7) |
| Glasgow Coma Scale | 80 (3.6) |
| **Hospital stay** | 61 (2.8) |
| **Intensive care unit stay** | 329 (15.0) |
| **Discharge time** | 60 (2.7) |
| **Outcome** | 56 (2.6) |
| **Onset year** | 10 (0.5) |

**Figure E1.** Prevalence of SAE in each hospital

Abbreviations: SAE, splenic artery embolization.

**Figure E2.** Comparison of prevalence trends for splenic artery embolization and open surgery among patients with blunt splenic injury

The number of patients who initially underwent SAE or open surgery is described in the graph.

Abbreviations: SAE, splenic artery embolization.

**References**

[1] Little RJA, Rubin DB. Statistical analysis with missing data. 3rd ed. New York: J. Wiley & Sons; 2019.
